# The Influence of Active Hamstring Stiffness on Markers of Isotonic Muscle Performance

**DOI:** 10.3390/sports9050070

**Published:** 2021-05-20

**Authors:** Sean P. Langan, Thomas Murphy, Wayne M. Johnson, Jadeon D. Carreker, Bryan L. Riemann

**Affiliations:** 1 Biodynamics and Human Performance Center, Armstrong Campus, Georgia Southern University, Savannah, GA 31419, USA; sean.langan@uconn.edu (S.P.L.); jadeoncarreker@gmail.com (J.D.C.); 2 Department of Electrical and Computer Engineering, Armstrong Campus, Georgia Southern University, Savannah, GA 31419, USA; tmurphy@georgiasouthern.edu; 3 Department of Mechanical Engineering, Armstrong Campus, Georgia Southern University, Savannah, GA 31419, USA; wmjohnson@georgiasouthern.edu

**Keywords:** sports medicine, modeling, musculoskeletal, biomechanics, tendon

## Abstract

Background: Previous research demonstrates hamstring muscle-tendon stiffness (HMTS) influences isometric strength, landing biomechanics and architectural tissue properties. However, the influence on kinetics & kinematics during other modes of strength testing (isotonic dynamometry) has yet to be established. Purpose: Investigate how HMTS influences kinetics and kinematics during a novel isotonic muscle performance test which has never been done for the hamstrings. Previous work using dynamometry has been limited to isometric or isokinetic contractions, so the novelty arises from our custom isotonic protocol which allows quantitative assessment of the stretch-shortening cycle. Methods: Twenty-six recreationally active individuals (15 males, 11 females, 23.8 ± 2.5 years) completed baseline testing for anthropometry and maximum isometric hamstring strength (MVIC). At least 48 h later, subjects completed a measure of HMTS (damped oscillation technique) followed by an isotonic knee flexion test (eccentric velocity 180°/s; concentric torque 25% of MVIC). Separate linear regression models with examination of residuals were conducted between HMTS and each muscle performance variable. Standardized coefficients determined the magnitude of the relationships. Results: Significance was found for all outcome variables tested. HMTS and rate of torque development demonstrated the strongest relationship followed by isotonic concentric peak torque. The weakest relationship observed was with isometric peak torque. Conclusions: These findings build off previous work quantifying HMTS by showing HMTS more strongly relates to dynamic versus static muscle testing and identifies the potential clinical utility of isotonic dynamometry.

## 1. Introduction 

Muscle and tendon collectively make up a unique functional unit at every joint, which is a biological development that has implications specifically tailored towards bipedal locomotion [1]. Although differences exist between muscle and tendon, a similar hierarchal structure exists in each, with previous research investigating the interaction of the respective structural components and their contributions to human movement [2,3,4]. Muscle-tendon stiffness (MTS) is an aspect of musculoskeletal function that is relevant to both athletic performance and injury risk. Tendon attaches to both bone and muscle, creating an impedance mismatch. Stiff muscle-tendon units are advantageous for performance via greater force transmission but can lead to muscle injury risk because decreased compliance predisposes excess strain. However, higher stiffness is always beneficial for ligament injury protection due to better joint stability, thus there is trade-off between performance and injury mediated by stiffness [5,6]. Stiffness can be divided into two distinct but interrelated components, active and passive. Active MTS is primarily determined by the series elastic and contractile components of Hill’s muscle model, such as myofibrillar cross-bridges and collagen orientations and cross-links within tendon [5,7]. The parallel elastic component (epimysium, perimysium, etc.) determines passive stiffness [8]. By modeling muscle-tendon units as springs, their active stiffness can be determined to quantify the ability to resist sudden joint displacement and tissue deformation [6,8], which is relevant to a muscle group such as the hamstrings which undergo a rapid stretch-shortening cycle during the swing phase of sprinting.

The hamstrings are indeed an attractive muscle group to investigate given their distinct role in running performance and injury susceptibility, and much attention has been paid to their function in the late swing phase of the stride cycle [9,10,11,12,13]. Epidemiological data clearly indicates hamstring injuries are frequent with high re-injury rates, thereby providing an impetus for their continued study [9,10,12,13,14,15]. Several sports place athletes at risk for such injuries [16,17,18,19,20], with linear high velocity running being the common denominator. Biomechanical modeling of elite athletes predicted hamstrings forces during sprinting to be approximately eight times the body weight and 40% greater than maximum isometric force [13]. Thus, there is a mechanical basis for the injury epidemiology coupled with the extreme loads in late swing providing clinicians with rationale for training and injury prevention programs. Whether these findings are simply a feature of all elite sprinters or can be explained by some morphological/functional characteristic of the muscle-tendon unit, such as active stiffness, is unclear since previous work has only addressed this characteristic in the context of isometric muscle testing and joint stability.

Previous findings have demonstrated that increased active hamstring stiffness (HMTS) strongly correlates with rate and amount of isometric force production, as well as less tibial translation during a drop landing [21,22]. These data suggest increasing HMTS may want to be targeted during training and rehabilitation programs. Isometric hamstring testing may indeed provide benefit in monitoring rehabilitation progress [23] as well as being a marker of sprint performance due to the positive relationship observed with sprinting speed [24]. However, the muscle-tendon performance insight provided by isometric testing may be limited as sport activity requires dynamic eccentric and concentric loading. It is perhaps the case that stronger, more clinically applicable relationships will be evident between HMTS and dynamic muscle performance testing, but these relationships have yet to be assessed to our knowledge.

Isotonic testing with a fixed computerized dynamometer may overcome the limitations of isometric testing since it allows for angular acceleration while providing a constant resistive torque under both concentric and eccentric conditions. There is a lack of literature employing isotonic dynamometry for research and clinical purposes, but recent work has demonstrated that at least for knee extension [25] and elbow flexion/extension [26], it is safe and reliable tool. It is reasonable to speculate that, compared to commonly used isometric and isokinetic dynamometry, isotonic assessment better resembles the conditions the muscle-tendon unit encounters during sport, particularly during eccentric-concentric transitions. As previously noted, it is during the late swing phase of sprinting when the muscle-tendon unit is experiencing high eccentric loading just prior to changing to concentric muscle actions, thus forcing the muscle-tendon unit resist rapid joint displacement and tissue deformation [6,8,27]. As stiffness is sensitive to changes in length of the muscle-tendon unit, quantifying active MTS and its role in various muscle testing procedures may be more appropriately approached by testing involving rapid length changes that expose the ability to quickly transition from eccentric to concentric contractions, a concept referred to as time-to-rebound in the plyometric literature [28].

HMTS can be manipulated through training [29], so demonstrating relationships between HMTS with kinetics and kinematics will have practical implications by modifying exercise prescription. Identifying performance variables (e.g., peak torque, rates of torque/velocity development, etc.) strongly related to HMTS would provide rationale to prescribe exercise with the goal of increasing or decreasing stiffness as needed. While the oscillation method of determining stiffness is fairly simplistic, it is predominantly a research tool not feasible for clinicians/coaches. In contrast, isotonic muscle performance testing can be more easily used either through fixed muscle dynamometry or using commercially available linear position transducers or accelerometers. By quantifying changes in the relevant isotonic variables as a result of exercise prescription, practitioners would have insight (albeit indirectly) regarding changes in the underlying HMTS. We hypothesized that those with higher levels of HMTS will demonstrate greater isotonic concentric peak torque (PT), rate of torque production (RTD), rate of velocity development (RVD), and a faster rebound time between eccentric to concentric muscle action transition. We also hypothesized that the relationship between HMTS and isotonic concentric peak torque will be greater than that of isometric peak torque. Therefore, the primary goal of this paper is to establish how active HMTS relates to kinetics and kinematics during an isolated dynamic isotonic dynamometry muscle performance test. We also sought to examine isometric peak torque since previous literature has demonstrated a clear connection with HMTS [22,30], and comparing the results of the new variables we introduce will provide insight into their accuracy.

## 2. Materials and Methods

### 2.1. Participants

Twenty-six healthy recreationally active subjects (15 males, 11 females; 23.8 ± 2.5 years; 174.2 ± 9.4 cm; 76.9 ± 12.6 kg) participated in this study. Subjects were recruited by word of mouth on Georgia Southern University’s Armstrong campus. Subjects were free from orthopedic, neurological, and chronic disease conditions within the past six months that would otherwise hinder their ability to run or complete isotonic testing, which they indicated on a medical history questionnaire and the 2019 Physical Activity Readiness Questionnaire (PAR-Q+), respectively. Additionally, participants were recreationally active defined as participation in some form (combination of moderate and vigorous intensity) of physical activity at least three to five days per week (30 min duration per session) based upon the American College of Sports Medicine guidelines [31]. This study was approved by Georgia Southern University’s Institutional Review Board and all data was collected in accordance with the Declaration of Helsinki. All subjects provided written informed consent before any data was collected.

### 2.2. Baseline Testing

Subjects started with a five-minute warm up by maintaining a cadence above 60 rpm on a cycle ergometer (50 W for females, 100 W for males), followed by dynamic lower body stretching. Next, participants completed an isometric strength test to determine the external load required for the stiffness assessment [22,30], as well as to establish the resistive torque for the isotonic dynamometry. The isometric assessment was conducted via maximal voluntary isometric contraction (MVIC) in 30° of knee flexion while lying prone on a fixed dynamometer (Biodex Medical Systems, Shirley, NY, USA). Subjects first performed a graded warm up (25%, 50%, 75%, 100% of perceived max effort) and were instructed to pull their leg towards themselves as if they were completing a leg curl. For the recorded trials, subjects were instructed pull their leg towards themselves/into the attachment as hard and as fast as possible until the investigator said to cease the contraction. Minor verbal encouragement was provided by the same investigator to all participants during the contractions, such as “keep going”, “as hard as you can”, “almost there”, etc. Three, five-second repetitions were completed with thirty seconds rest between repetitions, and the peak torque across the three trials was averaged and utilized for analysis.

### 2.3. Active Muscle-Tendon Stiffness

**Figure 1:**
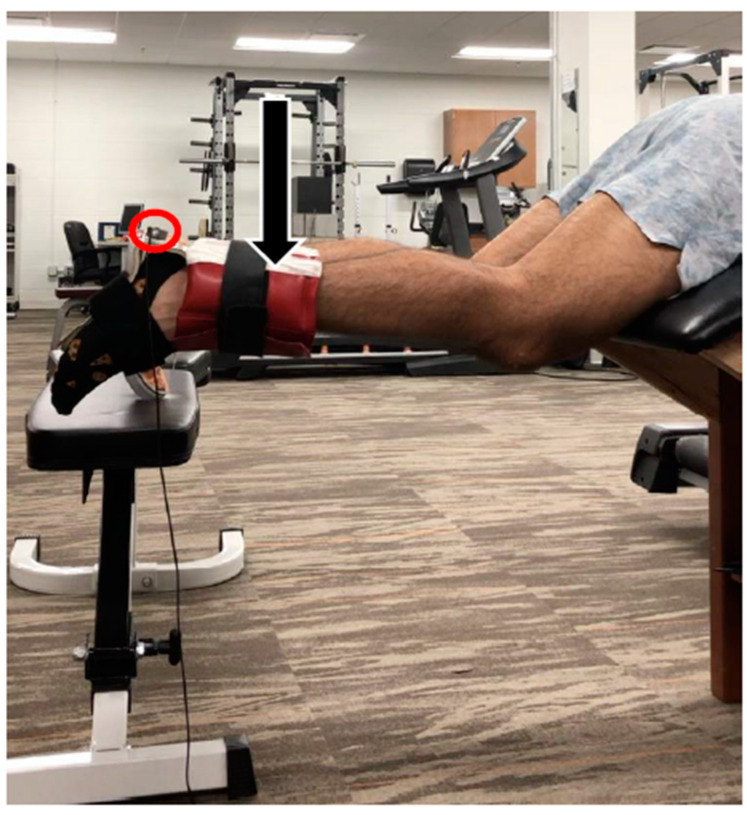
Experimental setup for assessment of active hamstring muscle-tendon stiffness. Subjects were fitted with a rigid ankle splint, a load corresponding to 25% MVIC, and a tri-axial accelerometer (circled in red). A member of the research team applied a downward manual perturbation (indicated by the downward black arrow) to obtain the damped oscillation which was viewed and analyzed offline.

The damping oscillation technique was employed to quantify active HMTS in an identical fashion to that of previous work (Figure 1) [8,21,22,30,32,33]. Subjects were positioned lying prone on a custom made plinth allowing simultaneous 30° hip and knee flexion as previously described by Blackburn et al. [32]. To avoid changes in ankle position affecting the length of the biarticular gastrocnemius, a rigid ankle splint held the angle joint in a neutral position. Twenty-five percent of the subject’s averaged isometric peak torque was applied to the ankle using cuff weights. Blackburn et al. has previously used 45% of MVIC, and 10% of subject body mass [8,22,30,32,33]. Pilot testing with the isotonic methods revealed 25% to be an optimal mode, thus 25% was used to maintain consistency between the isotonic testing and stiffness loads. While the tibia was held horizontal by the subject via isometric hamstring contraction, a manual perturbation was applied to the distal lower leg, causing a mild and transient stretch of the hamstrings. A tri-axial accelerometer (5 g, Dytran Instruments, Chatsworth, CA, USA) secured to the calcaneus conveyed the vertical acceleration of the shank-foot system. During each repetition, subjects were instructed to hold the shank as still as possible in the horizontal position and not to actively interfere with the perturbation, which was indicated by EMG signal on the medial and lateral hamstrings.

### 2.4. Isotonic Dynamometry

**Figure 2:**
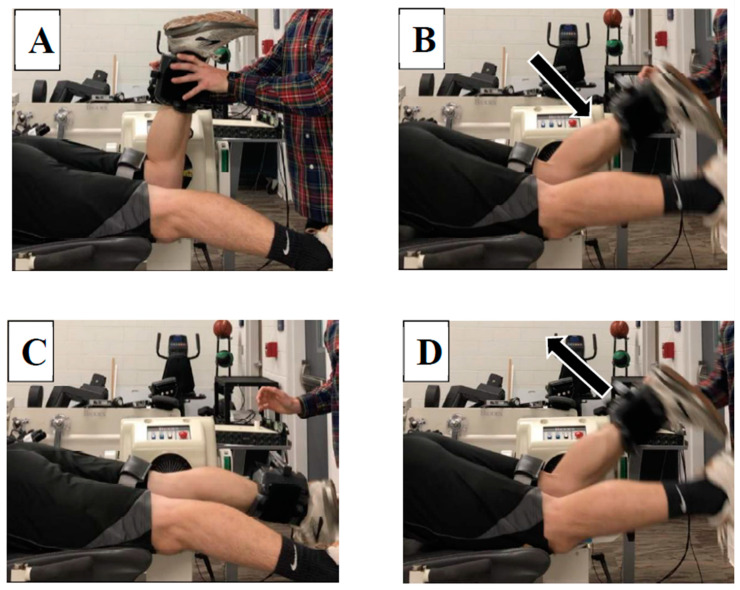
Step-by-step sequence of the isotonic hamstring test. (**A**) Participants began in 90° knee flexion; (**B**) eccentric knee flexion at 180°/s; (**C**) end range knee extension; (**D**) concentric knee flexion against a relative torque of 25% MVIC.

For the isotonic testing, participants were positioned prone in the fixed muscle dynamometer similarly to the isometric testing with straps placed on the thigh and waist. The test was then explained to the subject and five practice trials were completed to allow familiarization. Subjects were instructed to concentrically flex their knee by telling them to “pull up as hard and as fast as possible when you feel your leg straighten”. It was explained that the goal of the assessment was to determine how quickly they can bring their leg back up a bent position after it reached full extension. The test was eccentric/concentric beginning in 90° knee flexion. The eccentric velocity was 180°/s while concentric torque was set at 25% of subject’s average isometric peak torque. The sequence of events can be seen in Figure 2A–D. Subjects completed one set of five repetitions as torque, position, and velocity signals were recorded and streamed into the MotionMonitor acquisition software package (Innovative Sports Training, Inc., Chicago, IL, USA). The subjects were instructed to contract their hamstrings concentrically and flex their knee as fast and as hard as possible when they felt their knee reach full extension, similar to the stretch-shortening cycle that occurs during the mid-to-late swing phase of sprinting, albeit under much more controlled circumstances than actual sprinting.

## 3. Data Reduction

**Figure 3:**
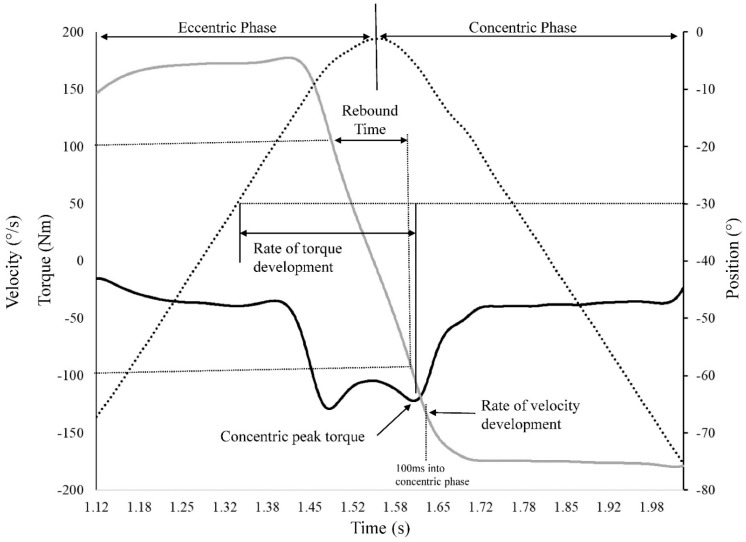
Graphical representation of the isotonic test computations from the torque (solid black line), velocity (solid gray line) and position (dotted line) to obtain concentric peak torque, rate of torque development, rate of velocity development, and rebound time.

During the isotonic and isometric trials, the analog torque, position and velocity signals from the Biodex were sampled at 1 kHz using the MotionMonitor acquisition software and saved for offline analysis. MATLAB (2019a) scripts (The Mathworks, Inc., Natick, MA, USA) were used to perform all muscle performance data reduction procedures. Based on the torque, position and velocity resolution settings of the Biodex, voltages were converted to Newton-meters, degrees and degrees per second. During the isotonic trials, the torque applied to the dynamometer by the participants was computed as:

(1) $$T_{Participant}=\left(I_{Foot-shank|knee}*\propto \right)+\left(I_{Attachment|knee}*\propto \right)-T_{Isotonic}-\;T_{Gravity}$$

where *T_Participant_* is torque applied by the participant to the dynamometer attachment, *I_Foot-shank|knee_* is the mass moment of inertia for the foot and shank about the knee joint, *I_Attachment|knee_* is mass moment of inertia of the Biodex attachment about the knee joint, α is the angular acceleration of the dynamometer shaft, *T_Isotonic_* is the isotonic torque applied by the Biodex and *T_Grav_*_ity_ is the torque associated with gravity acting on the foot-shank-attachment system. The mass moment of inertia for the Biodex attachment was determined by modeling the attachment in SolidWorks (Dassault Systèmes, Waltham, MA, USA). The mass moment of inertia for the shank and foot about the knee joint were determined based on Dempster parameters as reported by Winter [34]. Isotonic peak torque was the highest *T_Participant_* value attained during the concentric phase (Figure 3). Rate of torque development was defined as the time between when the knee extended past 30° during the eccentric phase and when peak torque during the concentric phase occurred. Rebound time was computed as the time interval/transition from a velocity of 100°/s during eccentric knee extension, to when they reached a velocity of 100°/s during concentric knee flexion. This can roughly be viewed as a surrogate for the proficiency of the stretch-shortening cycle by reflecting how quickly subjects can transition from an eccentric muscle action to a concentric muscle action.

Rate of velocity development was computed as the concentric velocity attained 100 ms into the concentric phase. Finally, after correcting the torque signal for gravity, isometric peak torque was computed as the highest attained torque value during the five-second trial. Both isotonic and isometric peak torques were normalized to body mass. Each variable was averaged across the three repetitions performed.

Accelerometer signals obtained from the damped oscillation assessment were sampled at 1 kHz into the MotionMonitor acquisition software and saved for offline analysis. The response to the perturbation initiates damping, and by calculating the oscillation decay amplitude and timing from the accelerometer signals, a measure of the muscle-tendon unit’s active stiffness was computed. The time between the first two dominant positive peaks of the acceleration after the perturbation was determined for each of the subjects using a MATLAB script and the damping frequency was determined using the difference between the times, f = 1/(t_2_ – t_1_). The times for the dominant positive peaks were also verified graphically. The average f of three trials for each subject (three subjects only had two trials) was used in the stiffness calculation: k = 4π^2^mf^2^, where k is the stiffness, m is the mass of the system (shank + foot + 25% load), and f is the oscillation frequency (Hz) [22].

## 4. Statistical Analysis

Exploratory data analysis for normality demonstrated stiffness, isotonic PT, and rebound time to be positively skewed. A natural logarithm transformation was used on these variables to obtain a normal distribution. Linear relationships between stiffness and isotonic PT, rebound time, RTD, RVD, and isometric PT were confirmed using scatter plots. Separate simple linear regression models were created to determine the magnitude of the relationships between stiffness and each muscle performance variable. By using a regression approach, the residuals could be examined for independence (Durbin-Watson test), normality (normal probability plots), homoscedasticity (partial plots), as well as for the presence of outliers (standardized residuals) or influential (Cook’s distances and covariance ratios) cases. The magnitude of the standardized coefficients were interpreted as moderate (0.3 to 0.5), large (0.5 to 0.7) and very large (0.7 to 0.9) [35]. All statistical analyses were performed with α < 0.05.

## 5. Results

**Figure 4:**
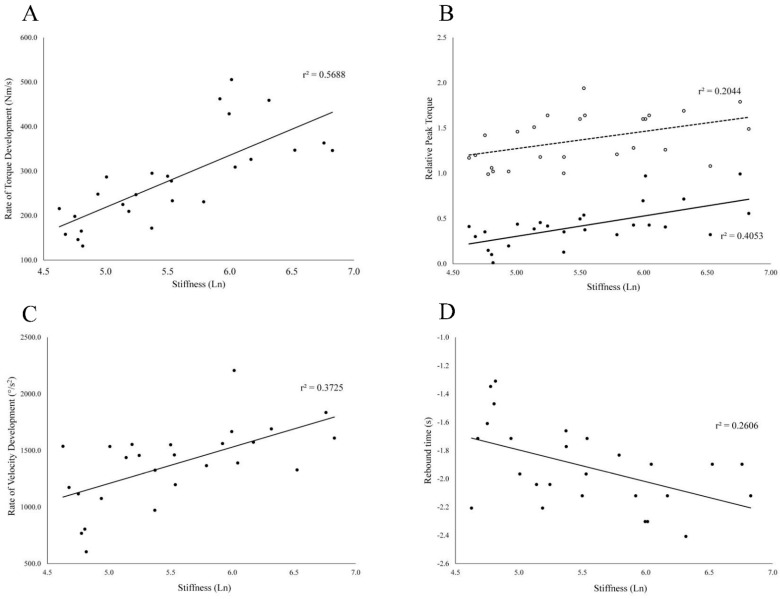
Scatter plots displaying natural log of hamstring muscle-tendon stiffness on the x axess and rate of torque development (**A**), relative peak torque (**B**), rate of velocity development at 100 ms (**C**), and rebound time (**D**) on the vertical axes. For the relative peak torques (**B**) the solid line and filled data points (•) represent isotonic peak torque, while dotted line and open data points (◦) represent isometric peak torque. It should be noted that isotonic values were log transformed due to normality violation and thus do not represent peak torque values in their original units. For rebound time, a more negative value represents a faster rebound time (faster transition between eccentric and concentric contractions).

All regression models were statistically significant (Table 1) with the standardized coefficient ranging in magnitude from 0.452 to 0.754. The regression diagnostics support the validity of each model Isotonic rate of torque development demonstrated the strongest relationship (Figure 4A) with active hamstring stiffness followed by isotonic peak torque (Figure 4B), rate of velocity development (Figure 4C), and rebound time (Figure 4D). Isometric peak torque demonstrated the weakest relationship with active hamstring stiffness (Figure 4B).

## 6. Discussion

The use of isotonic dynamometry is largely underutilized and there is limited work that have examined the effects of isotonic protocols, but it has been shown to be safe and reliable [25,26]. Compared to isokinetic and isometric dynamometry, isotonic testing includes angular acceleration, making it an attractive method that may bear more relevance to dynamic human movement. Isokinetic testing for the hamstrings in regard to identifying those with injury risk has demonstrated mixed results and therefore the utility of isokinetic testing remains questionable in certain circumstances [36,37,38]. In the current investigation, we were able to show that there are several significant relationships between active HMTS and our isotonic muscle performance test. Specifically, not only was peak torque related to HMTS, but also the three characteristics related to the eccentric-concentric transition time, and rates of concentric torque and velocity. These findings support the notion that enhanced HMTS may lead to improved dynamic muscle performance, which in turn may have implications for injury prevention. Our protocol attempted to reciprocate the late swing phase of running by employing the highest eccentric velocity allowed followed by a maximum concentric contraction. While we are not asserting that this a “functional” test due the velocities in sprinting being of much greater magnitude, we do believe it could provide clinically useful information not available from isometric or isokinetic testing. Further, equipment constraints limited our ability to fully replicate the joint positions in the late swing phase (hip flexion + knee extension). However, since active HMTS strongly related to isotonic concentric PT and RTD, it would be of interest to determine if these variables relate strongly to running performance and markers of injury risk, as has been previously shown albeit during an isometric hamstring assessment [24].

The outcome variables in the present investigation were chosen because of their potential relevance to translate to functional situations encountered during sport. RTD highlights the individual’s ability to rapidly produce torque during contraction. Ishøi and colleagues demonstrated that isometric hamstring RTD relates to sprint acceleration capacity in elite youth football players [24]. We sought to examine this variable during the isotonic test because although the previously mentioned isometric findings are intriguing, sport does not occur in a static environment. The ability to rapidly produce concentric torque in response to an eccentric change in length, as it does during isotonic testing (and sport), theoretically would bear more relevance to sprinting because of the high velocity eccentric-concentric transition that occurs in the late swing phase. Further, the enhanced stiffness-mediated RTD would reduce electromechanical delay, which has been shown to be twice as long than the antagonist quadriceps and suggested contribute to knee injury. RVD quantifies average angular acceleration during the period of interest (100 ms for the current study), and was shown to have good reliability during isotonic quadricep testing in older adults [25]. The importance of RVD is that it in addition to reflecting how quickly the muscle-tendon can produce torque, higher accelerations of the limb through a concentric muscle action would benefit performance. In many sports there is constant flux in acceleration and the individual’s ability to optimize how quickly they accelerate is crucial. As described previously, isokinetic and isometric testing do not include angular acceleration, thereby *requiring* the use of an isotonic test mode to quantify the velocities of the limb as a result of muscle actions. Finally, rebound time has been discussed previously in regard to medicine ball mass and the efficiency of the stretch-shorten component during plyometric throwing exercise [28]. Rebound time directly reflects the ability to reverse eccentric momentum and minimize the time between eccentric to concentric actions. In the current study, based on extensive pilot testing, we computed rebound time as the time between eccentric velocity at 100°/s to concentric velocity at 100°/s. A faster rebound time thus reflects a more efficient movement reversal and therefore more efficient utilization of strain energy stored during the eccentric phase. Based on the definition of muscle-tendon stiffness [6,22,30], we hypothesized that greater HMTS would be associated with an enhanced ability to reverse movement direction and stretch-shorten cycle efficiency. Our results confirmed this hypothesis.

Demonstrating a significant relationship between isometric peak toque and HMTS in the current study was expected as the work of Blackburn and others has consistently shown a link between active hamstring stiffness and isometric force production [6,21,22,30,33]. More importantly, the current study revealed a stronger relationship between HMTS and peak torque production when testing dynamically (i.e., isotonically) compared to the isometric mode. The magnitude of the relationship between HMTS and isometric peak torque (r^2^ = 0.204) was ~half the magnitude between HMTS and isotonic concentric peak torque (r^2^ = 0.405). Given the definition of active stiffness being sensitive to changes in tissue deformation and joint displacement [22,28,30], we hypothesized that incorporating a mode of muscle testing that exposes the muscle-tendon unit to length changes, as which occurred in the eccentric-concentric isotonic bout, stronger relationships would be observed. This is a novel discovery with practical ramifications because the isotonic test appears to expose to a greater magnitude the role active stiffness plays in dynamic muscle function. Based on the relationships reported by Ishøi and colleagues [24] there exists a relationship between isometric RTD and sprint performance; we speculate that when using isotonic testing these relationships will be accentuated. Fixed computerized dynamometry is often only used isokinetically in many clinical and research settings to gain insight into dynamic muscle performance. Utilizing the isotonic testing mode may elucidate relevant muscle performance characteristics to guide training.

The results of our work should be considered in the context of the inherent limitations of this study. The patient positioning platform associated with the fixed dynamometer prevented replicating hip flexion positions used during the stiffness assessment, and more importantly, the hip position during the terminal swing phase of running. Terminal swing elicits marked strain on the hamstrings as active hip flexion during knee drive stretches the proximal portion, and rapid angular acceleration of the shank as the leg swings beneath the center of mass eccentrically loads the distal portion simultaneously. Unfortunately, we were unable to reciprocate such strains in our isotonic test, which would have undoubtedly added valuable information. However, attempting to reciprocate the exact mechanics of a complex task such as sprinting during dynamometry is simply not feasible, but in our view, isotonic testing at least bears more relevance compared to other dynamometry modes that do not involve angular acceleration or stretch-shortening cycles. Future research building upon these results should consider a modified patient positioning platform to overcome this limitation. The gap between isolated muscle testing and functional activity will always remain but identifying novel protocols with the most translation to clinical practice will ultimately remain a goal. Our results suggest that active muscle-tendon stiffness influences dynamic function, but full confirmation of our concept needs to be addressed through proper HMTS intervention trials in active populations, and comparison with other traditional protocols.

We demonstrated several relationships between active stiffness of the hamstrings and measures of muscle performance during isotonic testing. Of these, an important finding is that stiffness had a stronger relationship with isotonic versus isometric peak torque, building off of previous work using isometrics. Our isotonic protocol also can give insight into aspects of muscle function not available during traditional methods, namely how the muscle-tendon responds to rapid changes in length. Similar protocols can allow clinicians to monitor relevant variables that give insight to muscle-tendon stiffness and thus guide exercise prescription.

## Figures and Tables

**Table 1:** Summary of the regression models predicting active hamstring stiffness from each of the isolated muscle performance characteristics.

Muscle Performance Characteristic	Coefficients		Standardized Coefficient	Significance
	Slope	Intercept		
*Isotonic*				
Peak torque	1.81	4.77	0.637	<0.001
Rate of torque development	0.005	4.16	0.754	<0.001
Rebound time	−1.16	3.30	−0.510	<0.001
Rate of velocity development	0.001	3.93	0.610	<0.001
*Isometric*				
Peak Torque	1.08	4.04	0.452	0.020

## Data Availability

The data presented in this study are available on request from the corresponding author. The data are not publicly available due to privacy.
